# GPs' attitudes towards and experiences of using the Dermabuddy health app for the management of patients with dermatological conditions: a descriptive cross-sectional study

**DOI:** 10.3399/BJGPO.2024.0038

**Published:** 2024-08-07

**Authors:** Caroline Burke, Karen Reidy, Paul Ryan, Aisling A Jennings

**Affiliations:** 1 Department of General Practice, University College Cork, Cork, Republic of Ireland; 2 Department of Dermatology, University Hospital Galway, Galway, Republic of Ireland

**Keywords:** dermatology, digital health, primary health care

## Abstract

**Background:**

Dermatological presentations are common in primary care. The digital health space is growing in investment, revenue, and in usership numbers. Doctors utilise mobile health apps for referencing, communicating, and for clinical decision making. Dermabuddy is a secure mobile health app by which information and expertise around skin problems can be shared among a group of medical professionals with the aim of finding the best treatment and management plan.

**Aim:**

To assess the utility of the Dermabuddy health app for GPs and associated trainees in the Republic of Ireland.

**Design & setting:**

This is a descriptive cross-sectional study, which involved a survey link distributed by email to GPs with Irish Medical Council membership.

**Method:**

GPs were surveyed on their experiences of using the dermatology mobile application, Dermabuddy.

**Results:**

In total, 203 members took this questionnaire (13.5% response rate). Ninety-six per cent who responded to the statement, *'The app was easy to use'*, agreed it was ‘easy’ or ‘very easy’. Eighty-seven per cent of those who responded to the statement, *'I would use this app again'*, agreed they ‘definitely would’. Fifty-eight per cent of those who responded to the statement, *'The app is useful for my healthcare practice'*, gave it a five-star rating. The content of 36 comments included advice for improvement and positive feedback.

**Conclusion:**

The Dermabuddy app was well received by participants in this study. Across all sections of the questionnaire looking at aspects of the app, including ease of use, interface and satisfaction, and usefulness, there was a positive response. Mobile health apps, such as Dermabuddy, may provide alternative solutions to meet the rising challenge of managing patients with dermatological conditions in primary care.

## How this fits in

Mobile health apps are utilised by doctors for various functions. There are limited studies looking at the use of apps for GPs that allow for peer discussion and learning, particularly in the area of dermatology. This study highlights the emerging area of technology in general practice. It shows an effective, well-functioning digital tool for the discussion of cases with peer support and specialist input is acceptable, desirable, and beneficial for GPs in the Republic of Ireland.

## Introduction

It is challenging to meet the current demand for dermatology care in general practice. Data from the Republic of Ireland show that at the end of 2022, 39 317 patients were awaiting a public dermatology outpatient appointment, and 20 900 of these patients were waiting >6 months.^1^ Patients living in lower socioeconomic classes and those who are geographically isolated, in particular, find it harder to access optimal health care.^2,3^ In relation to skin cancer, a disparity in melanoma outcomes for people of lower socioeconomic status has been shown, and in the treatment of acne those who are deprived are less likely to access Roaccutane treatment.^4–8^ Training and skillsets in dermatology in general practice are variable and GPs feel they are underskilled in some aspects of dermatology.^9^

Dermatological conditions have recognised negative effects on a patient's quality of life and functioning, and are associated with higher depressive symptoms, loneliness, and social isolation.^10^ Therefore, there is a need to find alternative solutions to meet the rising challenge of managing patients with dermatological conditions optimally in general practice today.

The digital health space, including mobile health apps, has been growing in recent years, with an 80% growth in dermatology apps in the period between 2014 and 2017, and the projected global market volume being USD 258.3 bn by 2029.^11,12^ Nearly one-third of dermatology apps are directed at healthcare providers.^13^ There is a variety of dermatology apps on the market, from wound management to real-time personalised sun protection advice, an app to help patients with psoriasis to adhere to their topical treatments, and an app for caregivers of children with atopic dermatitis.^14–17^ The World Health Organization's Global Observatory for eHealth defines mHealth as *’medical and public health practice supported by mobile devices‘*;^18^ mHealth applications include the use of mobile devices in collecting community and clinical health data, delivery of healthcare information to practitioners, researchers, and patients, real-time monitoring of patient vital signs, and direct provision of care.^19^

GPs are comfortable with technology and are willing to prescribe mobile health apps.^20^ Doctors already use mobile phones in the clinical setting for communication, to reference medical information, and to assist with decision making.^21,22^

Dermatology is an area where mobile apps have a particular role.^23^ Skin problems are the primary presenting issue in nearly 15% of consultations, with the top three conditions being eczema, infection, and benign tumours.^24,25^ Predicted modelling for diseases, such as non-melanoma skin cancers (NMSCs), shows disease burden is expected to rise by at least 1.5 times the current rates by 2044.^26^ Skin disease on a global level is the fourth cause of non-fatal disease burden and this is also an important issue in the ageing population.^27,28^

### The Dermabuddy application

The Dermabuddy application was set up in February 2020. It is a secure mobile health app by which information and expertise around skin problems can be shared among a group of other medical professionals via a mobile application with the aim of finding the best treatment and management plan for patients. Members must register to join the app and provide their name, Irish Medical Council number, email, and place of work. One can then use the app to view anonymised clinical details and images of dermatological presentations in primary care. Members can also use the app to seek advice from their peers on the diagnosis and management of clinical cases. At the time of writing, 1434 queries have been posted. The Dermabuddy app is free for healthcare professionals, and there is a privacy policy available on the website.

GPs with a special interest in dermatology support and contribute expertise to this secure discussion forum. There are no other mobile health apps (to the authors’ knowledge) that provide a closed peer discussion forum for dermatology cases in general practice.

This app was funded by a medical entrepreneur (a GP). In order to sustain the app and to pay for operational fees (hosting, user support, and other features), there is limited advertising offered to third parties, including pharmaceutical companies. The app is not designed to be a commercial offering but it does generate advertising revenue to cover the operational costs of the app. Clinical responsibility lies always with the doctor who posts the case, no matter what suggestions have been made, as per the terms and conditions that have been agreed by the doctor after signing up.

### Aims and objectives

The primary aim of this study was to assess the acceptability and utility of the Dermabuddy health app for GPs and GP trainees in the Republic of Ireland. The objectives are to gain insights into the attitudes and experiences of GPs and trainees with regard to ease of use of the app, the satisfaction levels with the app and its interface, and the usefulness of the app with regard to delivery of care, as a learning resource, for managing patient care and accessing services.

## Method

This is a descriptive cross-sectional study surveying GPs' experiences of using the dermatology mobile application, Dermabuddy. The study outcomes or end points are an understanding of GP attitudes to and experiences of using the Dermabuddy app, and to see whether there are any specific aspects of the Dermabuddy app that could be improved.

Inclusion criterion for the participants was that they were members of Dermabuddy, which is restricted to qualified doctors with current Irish Medical Council membership.

The mHealth App Usability Questionnaire (MAUQ) is a validated questionnaire developed in 2019 specifically for mobile health apps. It is customisable for patient or healthcare providers and to interactive or standalone apps.^29^ The MAUQ is specifically developed considering the unique properties of a healthcare app, and this validated tool was used to compose the online SurveyMonkey programme questionnaire.

Consent was sought in the first question, which was mandatory in order to access the survey. The following 18 questions in the questionnaire were based around the topics of ease of use and satisfaction, system information arrangement, and usefulness. Questions could be skipped and participants could opt out at any time. Each question was a statement and the options to answer were a mixture of yes or no options, star ratings 1–5, and Likert scales; for example, ‘very easy’ to ‘very difficult’. The last question was an optional free-text box for the purpose of feedback or comments.

The data controller for Dermabuddy sent the email with the participant information leaflet and survey link to members of the Dermabuddy app; in total, 1503 potential participants. The survey was open for participation between 1 September 2021 and 13 October 2021.

The study was reported according to the Strengthening the Reporting of Observational Studies in Epidemiology (STROBE) guidelines.^30^

## Results

In total, 203 out of 1503 (13.5%) participants consented to take the survey. The number of responses to the individual questions ranged from the maximum number, which was 173 responses (to the first statement) to the least number of responses, which was 151 responses (to the 4th last statement). Thus, there was not a sustained number of responses to the questions in the survey. There is no information available on the reasons for not answering all questions.

Across the section on ease of use and satisfaction, the app received positive responses on being easy to use (very easy, 62%; easy, 34%), easy to learn how to use (very easy, 66%; easy, 32%), the interface was well received and liked (84%), and the information on the app was well organised (68%). In response to the statement *'I would use this app again'*, 87% agreed they definitely would, and to *'Overall, I am satisfied with this app'*, 97% agreed (Table 1).

**Table 1.** Breakdown of 'Ease of use and satisfaction' survey responses.

Question	Response option	*n* (%)
The app was easy to use (*n* = 173)	Very easy	107 (62)
	Easy	59 (34)
	Neither easy nor difficult	6 (3)
	Difficult	1 (1)
	Very difficult	0 (0)
It was easy for me to learn to use the app (*n* = 172)	Very easy	113 (66)
	Easy	55 (32)
	Neither easy nor difficult	4 (2)
	Difficult	0 (0)
	Very difficult	0 (0)
I liked the interface of the app (*n* = 171)	Agree	144 (84)
	Neither agree nor disagree	24 (14)
	Disagree	3 (2)
The information in the app was well organised, so I could easily find the information I needed (*n* = 171)	Agree	116 (68)
	Neither agree nor disagree	40 (23)
	Disagree	15 (9)
I feel comfortable using this app in work settings (*n* = 172)	1-star rating: ‘Not so much’	3 (2)
	2-star rating	16 (9)
	3-star rating	28 (16)
	4-star rating	45 (26)
	5-star rating: ‘Absolutely’	80 (47)
The amount of time involved in using this app has been fitting for me (*n* = 170)	Yes	162 (95)
	No	8 (5)
I would use this app again (*n* = 172)	Definitely would	149 (87)
	Probably would	22 (13)
	Probably would not	1 (1)
	Definitely would not	0 (0)
Overall, I am satisfied with this app (*n* = 172)	Yes	167 (97)
	No	5 (3)

**Figure 1.**
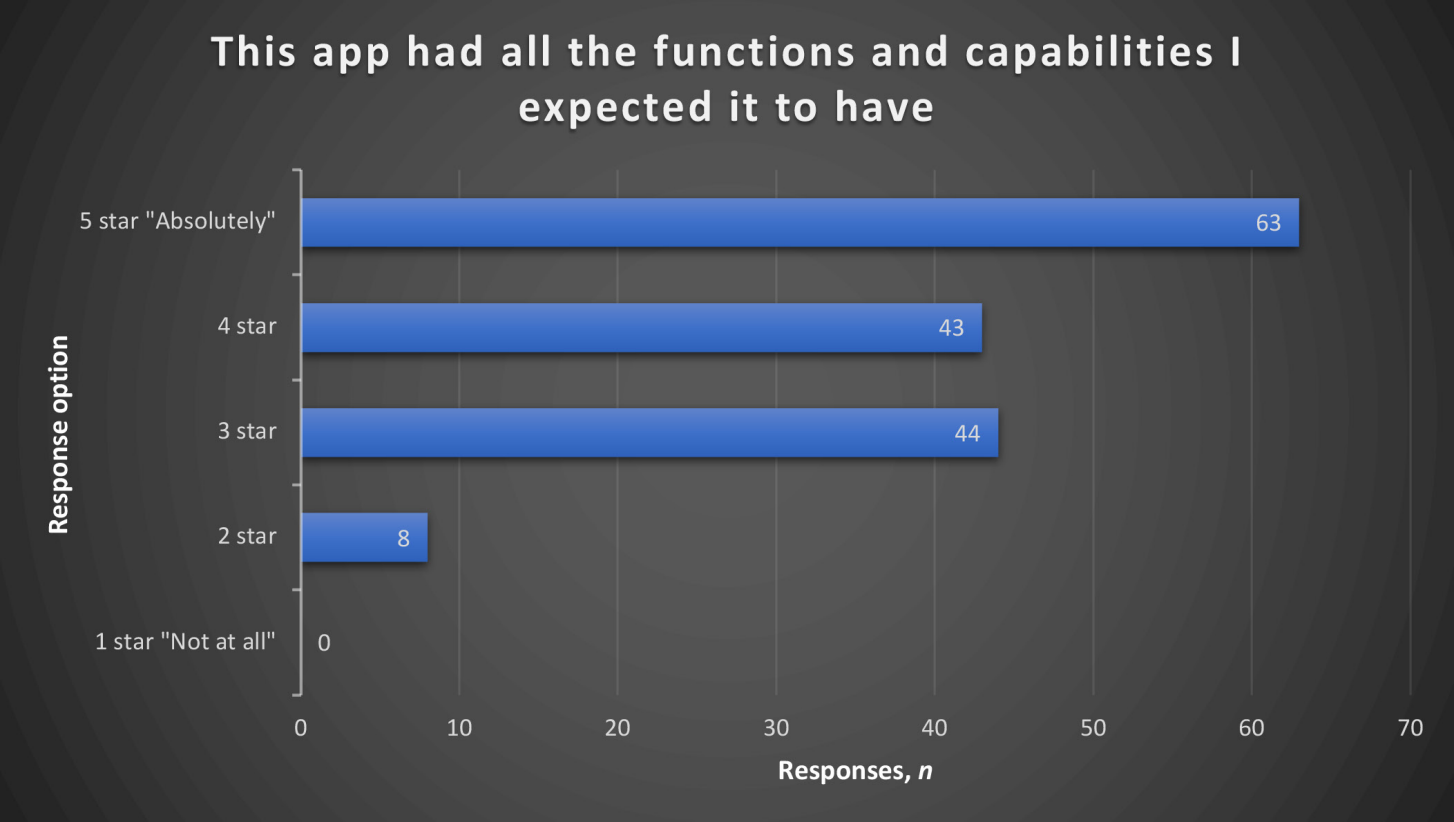
Responses to the statement *‘This app had all the functions and capabilities I expected it to have’*

In the section on system information, 81% agreed the interface functions were appropriate (Table 2). In total, 40% of respondees chose ‘Absolutely’ (a 5-star rating) in response to the statement *‘The app had all the functions and capabilities I expected it to have’* (Figure 1).

**Table 2.** Breakdown of 'System information arrangement' survey responses

Question	Response option	*n* (%)
Whenever I made a mistake on this app, I could recover easily and quickly (*n* = 158)	Agree	64 (41)
	Neither agree nor disagree	89 (56)
	Disagree	5 (3)
This mHealth app provided an acceptable way to deliver healthcare services (*n* = 158)	Agree	131 (83)
	Neither agree nor disagree	26 (16)
	Disagree	1 (1)
The app adequately acknowledged and provided information to let me know the progress of my action (*n* = 156)	1-star rating: ‘Not so much’	5 (3)
	2-star rating	13 (8)
	3-star rating	50 (32)
	4-star rating	33 (21)
	5-star rating: ‘Absolutely’	55 (35)
The navigation was consistent when moving between screens (*n* = 155)	Yes	143 (92)
	No	12 (8)
The interface of the app allowed me to use all the functions (such as entering information, replying to posts or messages, viewing information) offered by the app (*n* = 155)	Yes	125 (81)
	No	30 (19)
The app had all the functions and capabilities I expected it to have (*n* = 158)	1-star rating: ‘Not at all’	0 (0)
	2-star rating	8 (5)
	3-star rating	44 (28)
	4-star rating	43 (27)
	5-star rating: ‘Absolutely’	63 (40)

**Figure 2.**
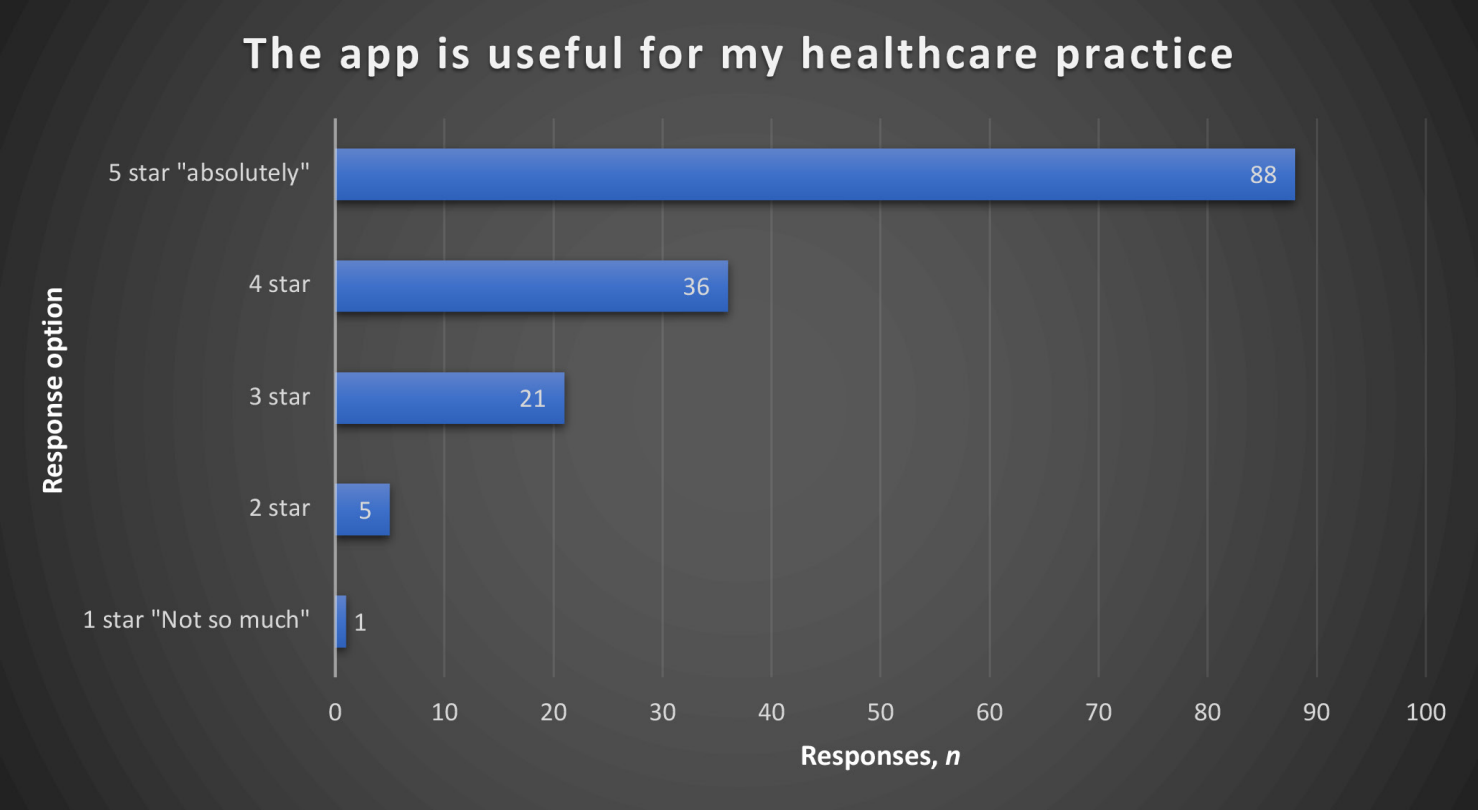
Responses to the statement *‘The app is useful for my healthcare practice’*

In the section on usefulness, the highest responses were to the statement on the mobile health app being an acceptable way to access educational material and track activities, with 63% in agreement. Fifty-eight percent of those who responded to the statement *‘The app is useful for my healthcare practice’* gave it a five-star rating; 29% of those who responded to the statement *‘The app improved my access to delivering healthcare services’* picked the ’a great deal’ option as a response. (Figures 2 3, Table 3).

**Figure 3.**
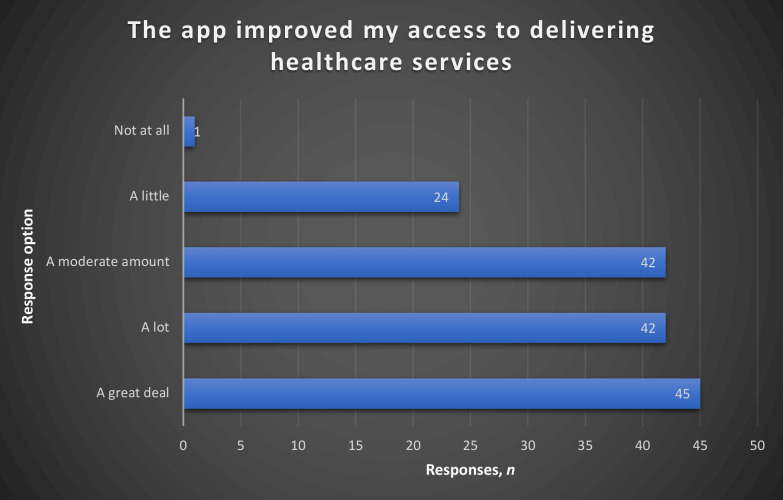
Responses to the statement *‘The app improved my access to delivering healthcare services’*

**Table 3.** Breakdown of 'Usefulness' survey responses

Question	Response option	*n* (%)
The app is useful for my healthcare practice (*n* = 151)	1-star rating: ‘Not so much’	1 (1)
	2-star rating	5 (3)
	3-star rating	21 (14)
	4-star rating	36 (24)
	5-star rating: ‘Absolutely’	88 (58)
The app improved my access to delivering healthcare services (*n* = 154)	A great deal	45 (29)
	A lot	42 (27)
	A moderate amount	42 (27)
	A little	24 (16)
	Not at all	1 (1)
The app helped me manage my patient's health effectively (*n* = 153)	1-star rating: ‘Not so much’	4 (3)
	2-star rating	9 (6)
	3-star rating	49 (32)
	4-star rating	34 (22)
	5-star rating: ‘Absolutely’	57 (37)
This mHealth app provided an acceptable way to deliver other healthcare services such as accessing educational materials and tracking my own activities (*n* = 153)	Agree	97 (63)
	Neither agree nor disagree	50 (33)
	Disagree	6 (4)

The content of the free-text responses could be divided into the following three themes: positive feedback; areas for improvement; and personal comments.

Positive feedback was provided in the comments section, such as one response:

*'This app has directly improved the care of over 20 of my patients who do not have private health insurance who would be waiting years to see dermatology as a public patient.'*

One user of the app described the app as:

*'... wonderful app — innovative learning tool, which is supremely useful when seeking a dermatology opinion quickly and reliably when unfortunately, due to referral times it can be difficult to get support clinically, given the current burden of care on secondary care dermatological services.’*

Positive feedback in terms of functionality included the comment:

*'... very easy to use, very informative even when cases not submitted by self.*’

One participant mentioned:

*'I don’t really use the app much. I feel a bit self-conscious contributing as you’re not anonymous and worried I’d be asking something really obvious.'*

The majority of comments were detailing areas for future improvement. Feedback in this domain spoke about the fact that posts weren’t always replied to and that there was a need for more expert input. For example:

*'Too many posts unanswered.'*

*'Sometimes people posted suggestions that are obviously wrong — this needs to be corrected.'*

*'Need more specialist input.'*

Technical and functional aspects of the app were commented on, particularly around the images aspect of the app, *'images loading crashing the app'*, and around posting a comment, *'sometimes a long comment is needed and when you try to go back you lose all of what you had typed'*.

A message system was suggested as a potential improvement, where an alert as a proposed function would help the user to know when a message was received.

A search option was also suggested as an improvement to the app, both as a way to search for rashes and diagnoses, and also as a way to search historical posts in terms of categories. The amount of time associated with using the current app seemed to be an issue:

*'... it can sometimes take a while to trawl back.'*

*'... historical questions could be categorised easier to find answers.'*

*'... improving the search functionality would be of help.'*

The learning aspect was also mentioned in the comments:

*'... need to try get people to circle back and tell us what happened with rash, diagnosis, or how responded to treatment*.*’*

A number of suggestions were provided by participants, including:

*‘Ability to create a list of learning cases or save your own cases.’*

*‘Easier access to the posts you made in the past.’*

*‘Don’t delete previous entries so we can search them months later.’*

## Discussion

### Summary

The app was well received across the three domains examined in this survey; that is, ease of use and satisfaction, system information arrangement, and usefulness.

The positive feedback in the free-text responses matched the sentiment of the responses to the individual survey statements. Comments also flagged the appropriateness to dermatology cases and how it improved patient care.

The free-text box responses give insight into general practice and some of the issues facing the specialty. Topics flagged referenced the need for structured advice, specialist input, the value of peer support, and the overall need for more resources. Reference is made to the two-tiered system, public patients waiting a long time, and the difficulty with referrals. The issue of time pressure on GPs was flagged. The sentiment of continuously striving for patients and how this resource matters to GPs was portrayed through a sense of comradery and gratefulness. The topic of continued learning and an appetite for reviewing and gaining more knowledge was flagged as a yearning for some users.

One person commented on the uncomfortable feeling of sometimes exposing a perceived lack of knowledge in a non-anonymous setting. This raises the psychological safety aspect within communities of practice, in which there may be an inhibition of some to post non-anonymous questions and this may be a limitation of the app, which in turn could affect the usership and thus the number of responses to this study survey. Psychological safety enhances a physician's engagement — perceived lack of knowledge and hierarchy are recognised barriers and the level of safety is related to the mode and format of performance feedback received.^31,32^ Hidden curriculum refers to the culture of the learning environment with regard to norms, values, and behaviours exhibited, and this is relevant in postgraduate medical education settings.^33^

### Strengths and limitations

The Dermabuddy members group is an unstudied cohort. As it is an anonymised survey, the risk of 'socially desirable responses' is eliminated.

Digital health and apps in general are an expanding area and this is the first study, to the authors’ knowledge, looking at the attitudes and experiences of Irish doctors in a general practice setting using a healthcare-related app to assist with management of patients with dermatological presentations.

This study may inform the current body of research on mobile health apps and their use for clinicians. It also gives specific quality improvement feedback on an active GP resource.

The survey is based on a validated tool for the evaluation of healthcare-related applications, but this tool has been validated only for the patient version of the app. The free-text box allowed for the collection of comments that would otherwise not have been picked up by the questionnaire alone — this allows for a broader view on the use and attitudes towards the app.

A small percentage of the overall number of members responded to the survey. There is no subscription fee and no renewal process is necessary for the app, so the number of active and recurrent users may be less than the number on the mailing list. There may be an element of selection bias — responders who have used the app and have found it to be beneficial or conversely who have found it to be unhelpful may be particularly enthusiastic to leave comments in the free-text section. There may be bias in that those who respond to the survey may have a particular interest in dermatology and it may not reflect the full picture in terms of usefulness, and all opinions of the app may not be collected.

This study evaluates a specific app used in the Republic of Ireland, and as the country has a uniquely functioning healthcare system this study may have a limited relevance to other healthcare systems from an international perspective.

This study did not collect information on frequency of use and sustained use of the application. The concept of adherence (incorporating the app into daily use or drop-outs from use) has been recognised as one of the problems and barriers related to the use of digital health applications.^34^ No data were collected on the demographics of the participants and this would be important in future research.

### Comparison with existing literature

There are many studies looking at healthcare app use among doctors internationally. For example, clarity, ease of use, speed, and support are deemed important app features in general.^35^ As the Dermabuddy app is a unique resource and is set up with both peer and specialist input for dermatological clinical cases on a secure forum, it is difficult to compare directly with other research studies on other physician-targeted healthcare apps. To the authors’ knowledge, no such studies have been performed on any similar apps in use by physicians.

Smartphones and mobile apps are used by physicians (including family physicians) in a clinical setting; 70% are used for communication and for education and/or training, and >50% preferred the use of smartphones and apps over other methods for contacting colleagues.^36^

Other apps, as tools that specifically target physicians that have been described, include a mobile clinical decision tool among emergency department (ED) clinicians in which the use of a mobile app version of the Ottawa rules was assessed for acceptability by 108 physicians. This study used a 23-question survey looking at usability and intention for further use, and was found to be favourably received.^37^ An app in Spain, which was developed with specialist input from rheumatologists for GPs, which helps support diagnosis and referrals by means of algorithms, has been described and is available for download. There is no user feedback on this app and the model for use is different as it is for referrals and not a forum for discussion of cases.^38^ A mobile health app assisting with diagnosis decision making in ophthalmology for primary care physicians in Spain (OpthalDSS) was evaluated by surveying 50 doctors who used the tool and 70% reported it performed in the expected way. Perceptions of doctors around the reliability of the information on the tool was also surveyed and found to be at 95%.^39^ A cross-sectional questionnaire study on 100 medical students in Cape Town published in 2018 showed that 65% access medical information on their phones, but there was no significant relationship between using mobile phone apps to access information and ensuring effective service to clients.^40^ A study performed in Germany, via a survey on 206 orthopaedic and trauma surgeons in 2018, reviewed their use of apps generally and 83% desired apps with intuitive usability, which were free to access.^41^ A survey to specifically assess an app as a clinical tool (Ortopex) in orthopaedics was performed in Brazil and used the system usability score, in which 90% of the 13 residents surveyed responded positively to the usefulness of the radiograph angle and distance measuring tool.^42^ Another app as a tool for physicians, DemPredict, was developed in Germany as a screening tool for Alzheimer's disease; a digital health compliance questionnaire was conducted and it was found that attitude towards technology was one of the main factors with regard to acceptability of use.^43^

Barriers to integrating apps into daily practice include lack of awareness of the apps, not seeing how they could help, not being confident in using mobile technology, time constraints, issues with hygiene and infection control, the distractibility aspect, and the effect on professionalism.^44–47^ Comfort with technology, age, and internet access may be factors that exclude certain groups from using mobile health apps.^48–51^

### Implications for research and practice

This study could prompt funding for digital tools in health care and inform further research on mobile health apps. Future applications in the area of dermatology could involve the triage and streamlining of patients from GP to dermatologists but this would need institutional support. Data were not collected on referrals saved owing to the use of the Dermabuddy application but the assumption can be made if this occurred then it may have implications for costs saved to the health system.

This study flags the lack of a current universal standard in terms of assessing the quality of mobile healthcare apps for physicians. This has been highlighted by Woulfe *et al* in the development of the Modified Enlight Suite, which is a framework to give healthcare professionals the means for evaluating apps they might recommend to patients (validated in an Irish setting).^52^

Many apps are not validated and lack expert input. There is a wide variety in terms of quality and, in one study looking at 131 medical diagnosis smartphone apps, the issue of risk of conflict of interest and presenting inaccurate information was raised.^53,54^ A study in 2016 showed that 81% of diabetes apps did not have privacy policies.^55^ Grundy *et al* in 2019 showed 79% of medicines-related mobile apps shared user data.^56^ Physicians are aware of the issues around access to raw data, data ownership, privacy and security and stated this as an ethical consideration when weighing up whether to use or prescribe an app.^57,58^ Physicians were more likely to incorporate apps into their usual practice if they had obtained a 'stamp of approval' and if there were assurances regarding safety and clinical effectiveness.^59^ In general mobile health apps are more likely to be utilised if they are convenient and user friendly.^60–62^

Since 2020 in Germany, GPs have the possibility to offer mobile health apps for patients on prescription and in 2022 in a study conducted via 96 qualitative interviews, GPs rated these digital health applications as favourable for their healthcare potential and deemed it useful that they were included in evidence-based guidelines.^63^

The reach of an app is not uniform or guaranteed (cultural aspects are relevant); further research is needed across all communities (Grundy *et al* in 2016 showed that 75% of descriptive studies on health-related app content have been conducted in English-speaking countries) for an inclusive and progressive integration of mobile health apps.^64,65^

In conclusion, this study shows an effective, well-functioning digital tool with peer support and specialist input is acceptable, desirable, and beneficial for GPs in the Republic of Ireland. It highlights quality improvement aspects for an active GP resource in the Republic of Ireland. It also gives an insight into the working and learning conditions and the needs and priorities of the contemporary Irish GP.

## Data

The dataset relied on in this article is available from the corresponding author on reasonable request.

## References

[1] The National Treatment Purchase Fund (2024). Outpatient waiting list..

[2] McCarthy S, Feeley K, Murphy M, Bourke JF (2019). Distance as a barrier to melanoma care. Ir Med J.

[3] Carmody K, Rouse M, Nolan D, Quinlan D (2020). GPs' practice and attitudes to initiating isotretinoin for acne vulgaris in Ireland: a cross-sectional questionnaire survey in primary care. Br J Gen Pract.

[4] Eriksson H, Lyth J, Månsson-Brahme E (2013). Low level of education is associated with later stage at diagnosis and reduced survival in cutaneous malignant melanoma: a nationwide population-based study in sweden. Eur J Cancer.

[5] Ortiz CAR, Goodwin JS, Freeman JL (2005). The effect of socioeconomic factors on incidence, stage at diagnosis and survival of cutaneous melanoma. Med Sci Monit.

[6] Pollitt RA, Swetter SM, Johnson TM (2012). Examining the pathways linking lower socioeconomic status and advanced melanoma. Cancer.

[7] Van Durme DJ, Ferrante JM, Pal N (2000). Demographic predictors of melanoma stage at diagnosis. Arch Fam Med.

[8] Moodie P, Jaine R, Arnold J (2011). Usage and equity of access to Isotretinoin in New Zealand by deprivation and ethnicity. N Z Med J.

[9] Le Roux E, Powell K, Banks JP, Ridd MJ (2018). GPs' experiences of diagnosing and managing childhood eczema: a qualitative study in primary care. Br J Gen Pract.

[10] Yew YW, Kuan AHY, Ge L (2020). Psychosocial impact of skin diseases: a population-based study. PLoS One.

[11] statista (2024). Digital health — worldwide..

[12] Flaten HK, St Claire C, Schlager E (2018). Growth of mobile applications in dermatology — 2017 update. Dermatol Online J.

[13] Ouellette S, Rao BK (2022). Usefulness of smartphones in dermatology: a US-based review. Int J Environ Res Public Health.

[14] Buller DB, Berwick M, Lantz K (2015). Smartphone mobile application delivering personalized, real-time sun protection advice: a randomized clinical trial. JAMA Dermatol.

[15] Svendsen MT, Andersen F, Andersen KH (2018). A smartphone application supporting patients with psoriasis improves adherence to topical treatment: a randomized controlled trial. Br J Dermatol.

[16] Xu X, Griva K, Koh M (2020). Creating a smartphone app for caregivers of children with atopic dermatitis with caregivers, health care professionals, and digital health experts: participatory co-design. JMIR Mhealth Uhealth.

[17] Biagioni RB, Carvalho BV, Manzioni R (2021). Smartphone application for wound area measurement in clinical practice. J Vasc Surg Cases Innov Tech.

[18] mHealth (2011). New horizons for health through mobile technologies.

[19] World Health Organization (WHO) (2018). Classification of digital health interventions v10: a shared language to describe the uses of digital technology for health..

[20] Della Vecchia C, Leroy T, Bauquier C (2022). Willingness of French general practitioners to prescribe mHealth apps and devices: quantitative study. JMIR Mhealth Uhealth.

[21] Nair AA, Afroz S, Ahmed BU (2021). Smartphone usage among doctors in the clinical setting in two culturally distinct countries: cross-sectional comparative study. JMIR Mhealth Uhealth.

[22] Byambasuren O, Beller E, Glasziou P (2019). Current knowledge and adoption of mobile health apps among Australian general practitioners: survey study. JMIR Mhealth Uhealth.

[23] Brewer AC, Endly DC, Henley J (2013). Mobile applications in dermatology. JAMA Dermatol.

[24] Le Roux E, Edwards PJ, Sanderson E (2020). The content and conduct of GP consultations for dermatology problems: a cross-sectional study. Br J Gen Pract.

[25] Kerr OA, Tidman MJ, Walker JJ (2010). The profile of dermatological problems in primary care. Clin Exp Dermatol.

[26] Hu W, Fang L, Ni R (2022). Changing trends in the disease burden of non-melanoma skin cancer globally from 1990 to 2019 and its predicted level in 25 years. BMC Cancer.

[27] Hay RJ, Johns NE, Williams HC (2014). The global burden of skin disease in 2010: an analysis of the prevalence and impact of skin conditions. J Invest Dermatol.

[28] Hay RJ, Fuller LC (2015). Global burden of skin disease in the elderly: a grand challenge to skin health. G Ital Dermatol Venereol.

[29] Zhou L, Bao J, Setiawan IMA (2019). The mHealth App Usability Questionnaire (MAUQ): development and validation study. JMIR Mhealth Uhealth.

[30] von Elm E, Altman DG, Egger M (2014). The strengthening the reporting of observational studies in epidemiology (STROBE) statement: guidelines for reporting observational studies. Int J Surg.

[31] Remtulla R, Hagana A, Houbby N (2021). Exploring the barriers and facilitators of psychological safety in primary care teams: a qualitative study. BMC Health Serv Res.

[32] Scheepers RA, van den Goor M, Arah OA (2018). Physicians' perceptions of psychological safety and peer performance feedback. J Contin Educ Health Prof.

[33] Torralba KD, Jose D, Byrne J (2020). Psychological safety, the hidden curriculum, and ambiguity in medicine. Clin Rheumatol.

[34] Giebel GD, Speckemeier C, Abels C (2023). Problems and barriers related to the use of digital health applications. J Med Internet Res.

[35] Hofer F, Haluza D (2019). Are Austrian practitioners ready to use medical apps? Results of a validation study. BMC Med Inform Decis Mak.

[36] Lee M, Bin Mahmood ABS, Lee ES (2023). Smartphone and mobile app use among physicians in clinical practice: scoping review. JMIR Mhealth Uhealth.

[37] Paradis M, Stiell I, Atkinson KM (2018). Acceptability of a mobile clinical decision tool among emergency department clinicians: development and evaluation of The Ottawa Rules app. JMIR Mhealth Uhealth.

[38] Urruticoechea-Arana A, León-Vázquez F, Giner-Ruiz V (2020). Development of an application for mobile phones (App) based on the collaboration between the Spanish Society of Rheumatology and Spanish Society of Family Medicine for the referral of systemic autoimmune diseases from primary care to rheumatology. Reumatología Clínica (English Edition).

[39] López MM, López MM, de la Torre Díez I (2017). mHealth App for iOS to help in diagnostic decision in ophthalmology to primary care physicians. J Med Syst.

[40] Achampong EK, Keney G, Attah NO (2018). The effects of mobile phone use in clinical practice in Cape Coast Teaching Hospital. Online J Public Health Inform.

[41] Dittrich F, Back DA, Harren AK (2020). Smartphone and app usage in orthopedics and trauma surgery: survey study of physicians regarding acceptance, risks, and future prospects in Germany. JMIR Form Res.

[42] Macedo FS de, Silva PG de B, Marçal E de BF, Rolim JPML (2021). Evaluation of usability, perception of usefulness, and efficiency of an application in interpreting imaging examinations and supporting decision-making in orthopedics. Telemedicine and E-Health.

[43] Schinle M, Erler C, Kaliciak M (2022). Digital health apps in the context of dementia: questionnaire study to assess the likelihood of use among physicians. JMIR Form Res.

[44] WHO (2019). Recommendations on digital interventions for health system strengthening..

[45] Zhang M, Bingham K, Kantarovich K (2016). Inter-professional delirium education and care: a qualitative feasibility study of implementing a delirium Smartphone application. BMC Med Inform Decis Mak.

[46] Katz-Sidlow RJ, Ludwig A, Miller S, Sidlow R (2012). Smartphone use during inpatient attending rounds: prevalence, patterns and potential for distraction. J Hosp Med.

[47] Imeri H, Desselle S, Hetemi D, Hoti K (2021). Mobile electronic devices as means of facilitating patient activation and health professional empowerment related to information seeking on chronic conditions and medications: qualitative study. JMIR Mhealth Uhealth.

[48] Bauer AM, Rue T, Keppel GA (2014). Use of mobile health (mHealth) tools by primary care patients in the WWAMI region Practice and Research Network (WPRN). J Am Board Fam Med.

[49] Hammerton M, Benson T, Sibley A (2022). Readiness for five digital technologies in general practice: perceptions of staff in one part of southern England. BMJ Open Qual.

[50] Carroll JK, Moorhead A, Bond R (2017). Who uses mobile phone health apps and does use matter? A secondary data analytics approach. J Med Internet Res.

[51] Kumar D, Hemmige V, Kallen MA (2019). Mobile phones may not bridge the digital divide: a look at mobile phone literacy in an underserved patient population. Cureus.

[52] Woulfe F, Fadahunsi KP, O’Grady M (2022). Modification and validation of an mHealth app quality assessment methodology for international use: cross-sectional and eDelphi studies. JMIR Form Res.

[53] Akbar S, Coiera E, Magrabi F (2020). Safety concerns with consumer-facing mobile health applications and their consequences: a scoping review. J Am Med Inform Assoc.

[54] Jutel A, Lupton D (2015). Digitizing diagnosis: a review of mobile applications in the diagnostic process. Diagnosis (Berl).

[55] Blenner SR, Köllmer M, Rouse AJ (2016). Privacy policies of Android diabetes apps and sharing of health information. JAMA.

[56] Grundy Q, Chiu K, Held F (2019). Data sharing practices of medicines related apps and the mobile ecosystem: traffic, content, and network analysis. BMJ.

[57] Conn J (2012). Most-healthful apps. Mod Healthc.

[58] Sarradon-Eck A, Bouchez T, Auroy L (2021). Attitudes of general practitioners toward prescription of mobile health apps: qualitative study. JMIR Mhealth Uhealth.

[59] Leigh S, Ashall-Payne L (2019). The role of health-care providers in mhealth adoption. Lancet Digit Health.

[60] Jacob C, Sanchez-Vazquez A, Ivory C (2020). Organizational, and technological factors impacting clinicians' adoption of mobile health tools: systematic literature review. JMIR Mhealth Uhealth.

[61] Kc B, Alrasheedy AA, Hing Goh B (2021). The types and pattern of use of mobile health applications among the general population: a cross-sectional study from Selangor, Malaysia. Patient Prefer Adherence.

[62] Nouri R, R Niakan Kalhori S, Ghazisaeedi M (2018). Criteria for assessing the quality of mHealth apps: a systematic review. J Am Med Inform Assoc.

[63] Wangler J, Jansky M (2023). Two years of approved digital health applications in Germany — perspectives and experiences of general practitioners with an affinity for their use. Eur J Gen Pract.

[64] Brewer LC, Fortuna KL, Jones C (2020). Back to the future: achieving health equity through health Informatics and digital health. JMIR Mhealth Uhealth.

[65] Grundy QH, Wang Z, Bero LA (2016). Challenges in assessing mobile health app quality: a systematic review of prevalent and innovative methods. Am J Prev Med.

